# “The cat and the calcium”. A case of delirium secondary to hypercalcaemia.

**DOI:** 10.1192/j.eurpsy.2023.2007

**Published:** 2023-07-19

**Authors:** T. Jiménez Aparicio, C. Vallecillo Adame, C. de Andrés Lobo, G. Medina Ojeda, M. Queipo de Llano de la Viuda, A. A. Gonzaga Ramírez, G. Guerra Valera, M. Fernández Lozano, M. J. Mateos Sexmero, B. Rodríguez Rodríguez, N. Navarro Barriga, M. A. Andreo Vidal, M. Calvo Valcárcel, P. Martínez Gimeno, M. P. Pando Fernández, I. D. L. M. Santos Carrasco, J. I. Gonçalves Cerejeira

**Affiliations:** 1 Servicio de Psiquiatría, Sacyl, Hospital Clínico Universitario Valladolid; 2 Servicio de Psiquiatría, Sacyl, Valladolid; 3 Servicio de Psiquiatría, SERMAS, Madrid; 4 Servicio de Psiquiatría, Sacyl, Palencia, Spain

## Abstract

**Introduction:**

Interconsultation with the psychiatry service is frequently requested from other specialties for the assessment and treatment of patients who present neuropsychiatric symptoms secondary to organic alterations. On the other hand (and in relation to this case), within the possible causes for the elevation of calcaemia figures, the most frequent are hyperparathyroidism and neoplasms, representing between these two entities 90% of cases (1).

Among the organic mental disorders, Delirium stands out, with an approximate prevalence between 1 and 2% (general population), which increases in hospitalized and elderly patients (2).

**Objectives:**

Presentation of a clinical case about a patient with delirium secondary to hypercalcemia, with hallucinations and behavioral disturbance.

**Methods:**

Bibliographic review including the latest articles in Pubmed about delirium (causes and treatment) and hypercalcaemia secondary to neoplasms.

**Results:**

We present a 52-year-old male patient, who went to the emergency room accompanied by his wife, due to behavioral alteration. Two days before, he had been evaluated by Neurology, after a first epileptic crisis (with no previous history) that resolved spontaneously. At that time, it was decided not to start antiepileptic treatment.

The patient reported that he had left his house at midnight, looking for a cat. As he explained, this cat had appeared in his house and had left his entire bed full of insects. His wife denied that this had really happened, and when she told the patient to go to the emergency room, he had become very upset.

As background, the patient used to consume alcohol regularly, so the first hypothesis was that this was a withdrawal syndrome. However, although the consumption was daily, in recent months it was not very high, and at that time no other symptoms compatible with alcohol withdrawal were observed (tremor, tachycardia, sweating, hypertension…).

We requested a general blood test and a brain scan. The only relevant finding was hypercalcaemia 12.9mg/dL (which could also be the origin of the previous seizure). It was decided to start treatment with Diazepam and Tiapride in the emergency room, with serum perfusion, and keep under observation. After several hours, the patient felt better, the hallucinations disappeared, and calcium had dropped to 10.2mg/dL. A preferential consultation was scheduled, due to suspicion that the hypercalcaemia could be secondary to a tumor process.

**Image:**

**Figure:**
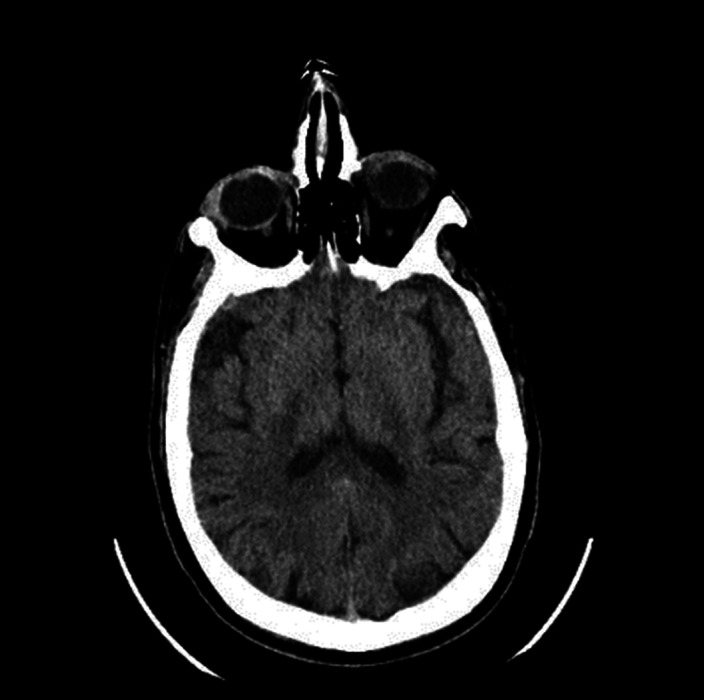

**Conclusions:**

It is important to rule out an organic alteration in those patients who present acute psychiatric symptoms. Hypercalcaemia is frequently associated with tumor processes (1) due to secretion of PTH-like peptide (4), so a complete study should be carried out in these cases.

Delirium has a prevalence between 1 and 2% in the general population (2).

Psychopharmacological treatment is used symptomatically, with antipsychotics (3). For the episode to fully resolve, the underlying cause must be treated.

**Disclosure of Interest:**

None Declared

